# Absence of mTOR Inhibitor Effect on Hepatic Cyst Growth: A Case Report of a Kidney Transplant Recipient with Autosomal Dominant Polycystic Kidney Disease

**DOI:** 10.1155/2012/513025

**Published:** 2012-12-13

**Authors:** L. Friedrich, F. Barbey, M. Pascual, J.-P. Venetz

**Affiliations:** Centre de Transplantation d'Organes, Centre Hospitalier Universitaire Vaudois, Avenue du Bugnon 46, 1011 Lausanne, Switzerland

## Abstract

Some experimental studies have suggested a beneficial effect of the mammalian target of rapamycin (mTOR) inhibitor use on hepatic and renal cyst growth in patients with autosomal dominant polycystic kidney disease (ADPKD). However, the results of clinical studies are conflicting and the role of mTOR inhibitors is still uncertain. We report the case of a patient with ADPKD who underwent deceased kidney transplantation because of an end-stage renal disease. The evolution was uneventful with an excellent graft function under cyclosporine (CsA) monotherapy. Some years later, the patient developed a symptomatic hepatomegaly due to growth of cysts. CsA was replaced by sirolimus, an mTOR inhibitor, in order to reduce or control the increase in the cyst and liver volume. Despite the switch, the hepatic volume increased by 25% in two years. Finally sirolimus was stopped because of the lack of effect on hepatic cyst growth and the presence of sirolimus side effects. The interest of our case resides in the followup by MRI imaging during the mTOR inhibitor treatment and 15 months after the restart of the initial immunosuppressive therapy. This observation indicates that mTOR inhibitors did not have significant effect on cyst-associated hepatic growth in our patient, which is consistent with some results of recent large clinical studies.

## 1. Introduction

The autosomal dominant polycystic kidney disease (ADPKD) is the most common hereditary kidney disease and is the fourth leading cause of end-stage renal disease in adults [1]. Studies outlined the role of mammalian target of rapamycin (mTOR) in the pathogenesis of renal and hepatic cyst formation. mTOR activity is upregulated in renal cyst lining epithelial cells in patient with ADPKD, suggesting a potential benefit of mTOR inhibitor treatment in reducing renal and hepatic cyst growth. However, no randomized study has been performed yet to evaluate the effect of mTOR inhibitors in reducing hepatic cyst growth in ADPKD patients. A slowing effect of mTOR inhibitors (or absence of slowing effect of mTOR inhibitors) on hepatic cysts growth still needs to be determined. A long followup is needed, because many cyst complications could change total kidney or hepatic volume. The adequate dose of mTOR inhibitor to inhibit the mTOR pathway with a minimum side effect as well as the adequate moment to initiate the treatment is also unknown and needs to be investigated. 

We report the case of an ADPKD patient who underwent a kidney transplantation and developed a progressive voluminous symptomatic hepatomegaly. Sirolimus, an mTOR inhibitors, was started because of preliminary data suggesting a potential benefit of mammalian target of rapamycin (mTOR) inhibitor therapy on hepatic cysts growth. But after two years of treatment, we did not observe any improvement in his condition and sirolimus was stopped.

## 2. Case Report

A 50-year-old man with autosomal dominant polycystic kidney disease (ADPKD) evolved to end-stage renal failure and hemodialysis was started in 1992. Because of voluminous kidneys, a right nephrectomy was performed in 1992, followed by the left one in 1994. In April 1998, a deceased donor kidney transplantation was performed in the right iliac fossa. Induction therapy consisted in seven days of polyclonal anti-T-cell globulin (thymoglobulin), because of delayed graft function. Maintenance immunosuppression consisted of cyclosporine (CsA) and prednisone. Steroids were withdrawn in December 1998. No acute rejection was observed. At one month, the serum creatinine was 130 *μ*mol/L and the glomerular filtration rate was estimated at 61 mL/min/1.73 m^2^ by MDRD. The serum creatinine remained stable during the followup and was of 113 *μ*mol/L in March 2011.

**Figure 1:**
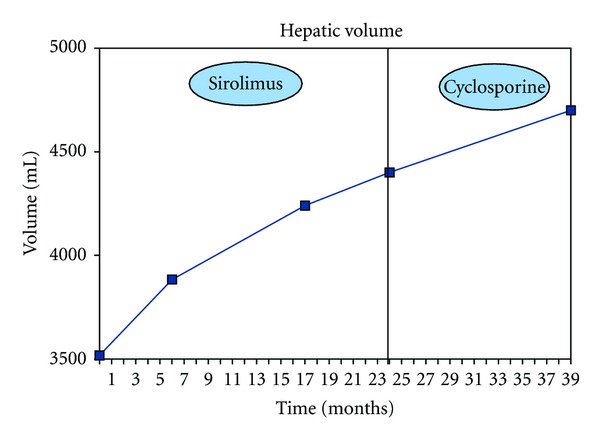
Hepatic volume since the initiation of mTOR inhibition therapy. Two-year followup by MRI. Month 0 = switch CsA to sirolimus. Month 24 = stop sirolimus because of absence of reduction growth of the hepatic volume and edema attributed to sirolimus.

During the followup in our outpatient clinic, the patient suffered from recurrent episodes of hepatic cyst infections, treated by antibiotics. In April 2007, bloating and abdominal discomfort developed. An abdominal MRI was performed showing a marked hepatomegaly with incomplete chronic Budd-Chiari syndrome, due to extrinsic venous compression by voluminous liver cysts. Because of worsening symptoms and many hepatic cyst infections, a new MRI was performed in June 2008, showing an increasing liver volume without progression of the Budd-Chiari syndrome. The liver volume was 3517 mL at this time. Because of preliminary data suggesting a potential benefit of mammalian target of rapamycin (mTOR) inhibitors therapy on hepatic cyst growth, CsA was stopped and sirolimus started at 0.5 mg/d to achieve serum levels between 4 and 6 ug/L (Table 1). MRIs were performed during the mTOR therapy because of worsening symptoms, and a followup was made with volumetric hepatic MRI. Only total hepatic volume was measured. Six months later, a new MRI showed a continuing increase in liver volume, which was estimated at 3883 mL. In November 2009, the liver volume was 4240 mL and in June 2010, 4400 mL (Figure 1). The chronic partial Budd-Chiari syndrome did not change.

Under sirolimus therapy, our patient did not report adverse events like cutaneous rash, diarrhea, and aphthous stomatitis, but he developed important legs edema. Hyperlipidemia was controlled with pravastatin and there was no significant proteinuria. Because of the continuing increase in liver volume (i.e., no significant effect of sirolimus) and the important discomfort due to bilateral legs edema, sirolimus was stopped and CsA restarted in June 2010. The differential diagnosis of leg edema was venous compression due to hepatomegaly. After restarting CsA therapy, the leg edema resolved, which was consistent with a sirolimus side effect. Fifteen months after sirolimus was stopped, a new MRI was done. The liver volume was at 4700 mL (Figure 1). The average hepatic growth over the two years of sirolimus therapy was of 37.5 mL per month versus 20 mL per month with cyclosporine.

## 3. Discussion

ADPKD is the most common hereditary kidney disease, affecting 1 in 400 to 1 in 10 000 births worldwide, and the fourth leading cause of end-stage renal disease in adults [1]. 

ADPKD is a genetically heterogeneous disease characterized by the development of cysts in the kidneys, liver, pancreas, seminal vesicles, and arachnoid membrane [1, 2]. Approximately 85% of cases are due to mutation in the polycystic kidney disease 1 gene (PKD1) in chromosome 16 and 15% to polycystic kidney disease 2 gene (PKD2) in chromosome 4 [3]. The glomerular filtration rate decline is associated with the increase in kidney volume [4]. Patients with PKD2 mutation have a later onset of disease and develop end-stage renal failure later than those with PKD1 mutation [1].

75–90% of cases develop a polycystic hepatic disease. It consists of proliferation and dilatation of biliary ductules and peribiliary glands [1]. Estrogens stimulate hepatic cyst cell proliferation, which explain why hepatic involvement is more prevalent and more severe in a young woman than in men. Polycystic hepatic disease is usually asymptomatic but symptoms can occur by mass effect or complications as cyst hemorrhage, infection, torsion, or rupture [1]. 

Studies outlined the role of the mammalian target of rapamycin (mTOR) in the pathogenesis of renal and hepatic cyst formation and growing, and experimental studies suggest mTOR inhibitors efficacy as treatment in ADPKD. mTOR is a kinase which integrates signals from cytokines, hormones, and growth factors. It coordinates cell growth, cell cycle progression, and proliferation. Human studies show that mTOR activity is upregulated in renal cyst lining epithelial cells in a patient with ADPKD. Recent studies have shown that somatostatin and mTOR inhibitors may have benefit hepatic cyst. Sirolimus, an mTOR inhibitor, is used as an immunosuppressive drug mainly in transplantation. Studies in rodent models show that mTOR inhibition retarded hepatic cyst expansion. A study in ADPKD patients after kidney transplantation suggested a reduction in kidney and hepatic cyst volume in patients treated with sirolimus compared to those treated with a calcineurin inhibitor [5]. However, Sirolimus blood levels to inhibit the mTOR pathway and avoid toxicity are not known [6]. Based on those findings, a randomized, cross-over study compared a 6-month treatment with sirolimus or conventional therapy alone on the growth of kidney volume measured in 21 patients with ADPKD and GFR equal or more than 40 mL/min [7]. 15 patients completed the study, 7 with sirolimus. Compared with the pre-treatment values, the posttreatment mean kidney volume increased less on sirolimus than on conventional therapy, but without significant differences. However, because of the small size and the short followup of the SIRENA study, no firm conclusions about the effect of mTOR inhibition could be reached. Recently, Serra et al. [8] randomized 100 patients with a GFR of at least 70 mL/min between sirolimus and conventional therapy and followed them during 18 months by MRI imaging. The conclusion was that in patients with ADPKD and an early stage of kidney disease, sirolimus did not stop the increase in kidney volume. Similary, Walz et al. [9] randomized 433 patients followed during two years to placebo versus everolimus, another mTOR inhibitor therapy. This study showed that everolimus did not significantly slow the increase of kidney volume during the first year, but the benefit was not maintained after the second year. Moreover, Canaud et al. compared two patients who received renal transplant from a donor with a known PKD1 mutation. One patient received steroids, tacrolimus, and mycophenolate mofetil as immunosuppression therapy and the other steroids, tacrolimus, and sirolimus. A five-year follow up showed no difference in cyst growth between the two patients [6]. No difference was noticed about the GFR between the two groups. Therefore, based on the current knowledge, the effect of the mTOR inhibition on progression of ADPKD remains uncertain.

The mTOR inhibition effect on hepatic cyst progression is unknown. Qian et al. [5] retrospectively examined 16 renal transplant patients with ADPKD disease who were randomized between sirolimus-mycophenolate mofetil-prednisone therapy and tacrolimus-mycophenolate mofetil-prednisone therapy in another trial. After an average of 19.4 months of treatment, the sirolimus regimen showed a reduction in polycystic hepatic volume compared with tacrolimus, but because of the small size, the retrospective form, and the short followup of the study, no conclusion could be made about the mTOR effect on hepatic volume progression in an ADPKD patient. Recently, He et al. [10] published a meta-analysis of randomized controlled trials about the efficacy and safety of the mTOR inhibitor therapy in patients with early ADPKD. Four studies were included, and analysis of total kidney volume, cyst volume, parenchymal volume, glomerular filtration rate, and adverse events was made. They concluded that short-term mTOR inhibitor therapy may slow down the increase in kidney volume and is relatively safe but that the impact on the GFR was limited. Long-term efficacity and safety are still unknown. The adequate moment to initiate the mTOR inhibitors therapy as well as the dose of the medication needs to be investigated. Novali et al. [11] used animal models of polycystic kidney disease (PKD) to evaluate the efficacy of low-dose levels versus high-dose levels of sirolimus on renal cyst growth. They also compared early and late initiation of the treatment. They concluded that high dose of sirolimus given in an early stage of the disease reduced significantly cyst formation in ADPKD mouse models, where low-dose levels do not. When initiated at a more advanced stage, low- and high-dose levels of sirolimus have not significant effects on cyst formation. The high doses used in mouse models are 10 to 20 times higher than those used for immunosuppressive therapy after transplantation, beyond the levels tolerated by humans.

Side effects were reported in the different studies. Most of them were controlled with medical treatment. Classical adverse events are aphtous stomatitis, rash, peripheral edema, diarrhea, hyperlipidemia, proteinuria, and infection.

We reported the case of a patient in which we introduced sirolimus to achieve serum levels between 4 and 6 ug/L late in the disease progression. Our case raises several questions concerning the use of mTOR inhibitors: when should the treatment be introduced, which serum should be targeted, and is it effective and safe? In our patient, mTOR inhibitor did not improve his abdominal condition and induce important legs edema. Hepatic cysts epithelia express a high level of mTOR suggesting that mTOR inhibitors may have an effect in downregulating hepatic cyst growth [5], but no randomized study has been performed yet to evaluate the effect of this therapy. Randomized studies are needed to determine the efficacy of mTOR inhibitors on hepatic cyst growing. A long followup is needed, because many cyst complications could change the total kidney or hepatic volume. The adequate dose of sirolimus to inhibit the mTOR pathway with a minimum of side effect [6] as well as the adequate moment to initiate the therapy is still unknown and needed to be investigated. The mTOR inhibitor effect on hepatic cysts growth still needs to be determined. 

## Figures and Tables

**Table 1:** Serum level of sirolimus during treatment.

Date of dosage (year/month/day)	Dose (mg/d)	Sirolimus level (ug/L)
08.10.15	0.5	5.6
08.12.03	0.5	4
09.01.21	0.5	6.2
09.02.27	0.5	5.1
09.07.08	0.5	5.6
09.09.16	0.5	4.2
09.09.30	0.5	3.7
09.09.10	0.5	2.8
09.11.04	1	10.5
09.11.18	0.5	4.3
10.01.22	0.5	3
10.05.12	0.5	5.3
10.06.02	0.5	3.1
10.06.23	0.5	3.8
